# Exploring implementation and sustainability of models of care: can theory help?

**DOI:** 10.1186/1471-2458-11-S5-S8

**Published:** 2011-11-25

**Authors:** Della A Forster, Michelle Newton, Helen L McLachlan, Karen Willis

**Affiliations:** 1 Mother and Child Health Research, La Trobe University, 215 Franklin Street, Melbourne, Victoria 3000, Australia; 2 Royal Women’s Hospital, Grattan Street, Parkville, Victoria 3052, Australia; 3 School of Nursing and Midwifery, La Trobe University, Bundoora, Victoria 3086, Australia; 4 School of Sociology and Social Work, University of Tasmania, Hobart, Tasmania 7001, Australia

## Abstract

**Objective:**

Research on new models of care in health service provision is complex, as is the introduction and embedding of such models, and positive research findings are only one factor in whether a new model of care will be implemented. In order to understand why this is the case, research design must not only take account of proposed changes in the clinical encounter, but the organisational context that must sustain and normalise any changed practices. We use two case studies where new models of maternity care were implemented and evaluated via randomised controlled trials (RCTs) to discuss how (or whether) the use of theory might inform implementation and sustainability strategies. The Normalisation Process Model is proposed as a suitable theoretical framework, and a comparison made using the two case studies – one where a theoretical framework was used, the other where it was not.

**Context and approach:**

In the maternity sector there is considerable debate about which model of care provides the best outcomes for women, while being sustainable in the organisational setting. We explore why a model of maternity care – team midwifery (where women have a small group of midwives providing their care) – that was implemented and tested in an RCT was not continued after the RCT’s conclusion, despite showing the same or better outcomes for women in the intervention group compared with women allocated to usual care. We then discuss the conceptualisation and rationale leading to the use of the ‘Normalisation Process Model' as an aid to exploring aspects of implementation of a caseload midwifery model (where women are allocated a primary midwife for their care) that has recently been evaluated by RCT.

**Discussion:**

We demonstrate how the Normalisation Process Model was applied in planning of the evaluation phases of the RCT as a means of exploring the implementation of the caseload model of care. We argue that a theoretical understanding of issues related to implementation and sustainability can make a valuable contribution when researching complex interventions in complex settings such as hospitals.

**Conclusion and implications:**

Application of a theoretical model in the research of a complex intervention enables a greater understanding of the organisational context into which new models of care are introduced and identification of factors that promote or challenge implementation of these models of care.

## Introduction

Maternity care provision in Australia has undergone many changes in recent years, and over time a variety of models of care provision have been developed and implemented. This is particularly the case in the public maternity care setting, in which around two thirds of Australian women receive care [1,2]. The impetus to introduce these new models is likely to be multifactorial, and ‘driven’ by different stakeholders, i.e. consumers, policy makers and care providers. It is also likely that the ‘outcomes’ of various models of care may be viewed differently by different groups; that is, an outcome of care highly valued by one group may not be valued by another. For example, for some, ‘safety’ (e.g. intervention rates, maternal and neonatal morbidity and mortality, and longer term health outcomes) might be of most value, whereas others might value consumer satisfaction, cost, staff satisfaction and recruitment/retention more highly.

There is evidence that in addition to safe maternity care, women want choice, control and continuity, and that increasingly, consumers of maternity services want greater access to midwifery led models of care and the opportunity to know their caregiver [3-6]. Although successive state-wide reviews of new mothers in Victoria have found that the model rated most highly by women is private obstetric-led maternity care, midwife-led models are also rated favourably [7]. When thinking about these findings, it is important to describe the Australian context. Two largely separate options for maternity care exist in Australia – public and private, with around two thirds of women accessing public care [1]. The private sector is characterised by obstetric-led care, whereas standard (or conventional) public maternity care incorporates a range of different approaches to care. In Australia, as in many developed countries, public maternity care has traditionally been fragmented, with different groups of caregivers providing care at different stages. In a typical example, a woman may have her antenatal care provided by one or a number of medical practitioners or midwives, and the care may be hospital or community based. In standard care models, labour and birth care is often provided by a midwife unknown to the woman and in more than 95% of cases takes place in hospital [6]. Following birth, women are usually cared for by another group of midwives in the postnatal ward, then after discharge yet another midwife/s may visit the woman’s home to provide care [8,9]. It may be that the private obstetric models have been successful in offering continuity of carer to women thus increasing satisfaction, while midwifery-led models of care have been challenged by barriers in providing primary carer models.

From a policy perspective, continuity of care has been strongly recommended throughout Australia [6] . In June 2004 the Victorian Department of Human Services (DHS) released a policy document “Future directions for Victoria’s maternity services” [5] which endorsed and promoted the expansion of public models of maternity care that offer continuity of *carer* . While the distinction between continuity of care and continuity of carer is not always clear [10], in general it is considered that continuity of *care* refers to a continuity of philosophy among a group of care providers, with a shared view of how care should be provided, or adherence to set guidelines and protocols [11,12], whereas continuity of *carer* implies care provision by a known provider [9,10] or fewer care providers [10,13]. Recently there has been an emphasis on continuity of *carer* as opposed to continuity of *care*[5,13], despite the supportive evidence (increased satisfaction, decreased interventions) for models of care such as team midwifery, that offer continuity of care rather than carer [13-15].

A number of studies report that midwives want to work in models that offer the opportunity to work autonomously and to provide continuity to women across the continuum of maternity care [16-18], and that this opportunity may be an effective retention strategy for such midwives [9,19]. However, not all midwives want to work in this way [9,19,20], particularly where the model involves working on call [9,21,22]. It is therefore critical that new models are properly evaluated with regard to midwife job satisfaction, recruitment and retention, [22] both for midwives providing continuity of care/r and those working in standard care [9]. Brodie reflects on the lack of research on the experiences of midwives and a lack of ‘sociological analysis of the implementation or maintenance of this innovation from their perspective’ [23] (p132), and suggests this is crucial, given that the main reason cited in the literature for the discontinuation of new models is midwife dissatisfaction [24].

There are complex factors involved in the introduction of new models of maternity care; organisations are complex, and new models may be impacted by internal and external factors. New models of care are likely to involve not just a single change, but multiple changes within both care provision and organisational structures. In light of this complexity, new models of maternity care fit the UK Medical Research Council (MRC) definition of complex interventions, in that they comprise multiple and inter-linking strategies that attempt to take a ‘whole of problem’ approach to health issues [25,26].

Complex interventions in health are those that are not limited to a single dose or activity, but comprise many potentially ‘active ingredients’ [27]. It is therefore critical that the impact of new models of care are rigorously evaluated, considering outcomes for women and infants as well as outcomes for midwives and other maternity care providers [9,22]. The capacity and willingness of organisations to implement and support a new model of care must also be considered. Thus, when researching new models of care, the ‘success’ of a randomised controlled trial (RCT) is only one factor involved in whether a new model of care will be implemented at the conclusion of the study or in other settings. We propose that there may be a number of other barriers and enablers to the successful implementation of new models of care which may account for the success or dissolution of such models, even when in the context of trials with positive clinical findings.

The aim of this paper is to discuss how theory can be used to explore, understand and interpret implementation strategies and the impact of organisational context when evaluating new models of health service delivery. We propose that consideration of these issues is crucial to considering sustainability for interventions that are shown to have beneficial outcomes. We use two case studies to illustrate the discussion, a retrospective reflection on an RCT which, despite positive findings, was not implemented; and a recent RCT that has used a theoretical framework to reflect on implementation of a new model within the trial context. The conceptualisation and rationale leading to the use of the Normalisation Process Model in exploring the implementation and organisational context of the second case study is discussed.

## The normalisation process model and theory

The aim of social theory is to provide a structured interpretation of the issue being investigated [28]. Social theory can provide a lens through which we can develop an understanding of the issue and thus design research that adequately explains and predicts how various factors interact and influence the outcome of, in this case, new models of care. When we were looking for a suitable theoretical framework to help in our understanding of how complex interventions are best implemented, evaluated and sustained within organisations such as health services, there were a number of sociological theories that had some applicability, e.g. theories around organisational change and planned behaviour, and others that address the issues of uptake and implementation of evidence.

We initially explored the possibility of using the Theory of Planned Behaviour [29] as a theoretical framework, given the type of intervention that was being evaluated; i.e. the intervention involved delivery of care by individuals (who may provide care in different ways) to a specific group of women. In examining this theory it became apparent that we would need to isolate the ‘behaviour’ or ‘behaviours’ that would be changed by the caseload role, or that could impact on delivery of care. While there may have been value in using this theory to examine modal salient beliefs (i.e. the commonly held beliefs in the research population) we speculated that moving from a traditional midwifery role to working in caseload involves a number of changes, not just in midwives’ behaviour, but in many aspects of their work and personal lives. Given that we wanted to give the participants the opportunity to identify what actually did change in their role, we decided that the Theory of Planned Behaviour may have been too restrictive, and also that it might not reflect the complexity of the implementation of a new model of maternity care, and the changes experienced by the midwives and the organisation as implementation was undertaken.

We similarly considered other theoretical approaches, however after much consideration, the sociological model that seemed to provide the best ‘fit’ in terms of the activity of implementation of a complex model of care into existing maternity services was The Normalisation Process Theory developed by May and colleagues. [30] This theory has a focus on exploring how a change of ‘work’ within an organisation such as a health service is implemented, and the complex interactions that need to be in place to ensure the embedding of change to allow sustainability. The theory proposes that ‘there is a common core to the work (implementation) of making practices routine elements of everyday life (embedding), and sustaining embedded practices in their social contexts (integration)’ [30] (p 538).

Normalisation Process Theory is a conceptual tool used primarily to examine the implementation of complex interventions in the clinical health care setting, particularly reflecting on how the changes become ‘normalised’ or accepted within institutions, demonstrated by changes in thinking, actions and organisation [30]. The authors of the theory are particularly interested in the work that people do, and how a complex change, such as a new model of care, becomes integrated within work practices [31]. May et al. describe it as a theory that can be used in prospective process evaluations of the introduction of complex interventions. Normalisation Process Theory assists in directing research to the questions that are intrinsic to sustainability, and focuses attention on implementation as a social process and on the social factors that may constrain or enhance the work of implementation.

The Normalisation Process Theory evolved from the Normalisation Process Model [30], in which the authors proposed that using a theoretical model would enable the identification of the conditions required to support the introduction of a complex intervention [32]. It is this model that has been used to frame elements of the evaluation of the second case study presented here. The model provides a framework on which to examine the facets of a complex intervention. A change such as the introduction of a model of care within an organisation is expected to impact in many ways, including alteration of resource allocation, changes to roles and responsibilities within the workplace, a changed perception of the service by those that use it and possibly a different way of working. The Normalisation Process Model (which was what was available at the commencement of our research, i.e. prior to the development of the theory) focuses on four constructs to allow for a closer examination of elements that are important in the embedding of a complex change [32]. *Interactional workability* relates to how the work is (or may be) different and how individuals need to work differently in a new model of care – the change required and how this impacts on others both within the service, and those that use the service. *Relational integration* relates to how the work is understood, and explores the shared understanding of the change of work allocation within a new model of care, the expertise required for any new roles, and employees’ beliefs about who is appropriate to undertake the work. *Skill set workability* – the place of work in the division of labour – explores who is responsible for the work, what skills, knowledge and attributes each contributes, and the agreed operational governance. *Contextual integration* explores how organisational sponsorship and control of work allows the new model to operate within the organisation, including the allocation of resources [32].

## Case studies and context

Two case studies provide the opportunity to reflect on the implementation of two midwifery models of care at the Royal Women’s Hospital (the Women’s), a tertiary hospital in Melbourne, Australia, and consider how we can use Normalisation Process Theory to understand the barriers and enablers to sustainability of new and complex models of care within maternity care settings. Both models were developed and implemented in the context of an RCT at the Women’s and neither had been in place in this setting prior to the trial. In both instances the introduction of these models was based on previous (limited) RCT evidence, particularly within the Australian context [33]. One tested *team midwifery* compared to usual care and the other tested *caseload midwifery* compared with usual care. These models are briefly defined here, as is ‘standard’ or ‘usual’ care.

During both trials, midwives’ clinic as well as hospital-based medical care options existed, and seeing a community-based general practitioner was also an option for antenatal but not intrapartum or postnatal care. There were various levels of continuity of care available to both women and midwives, although no model offered continuity of *carer* across the childbearing continuum. A birth centre was also an option of care for women during the team midwifery trial and afterwards, however this model ceased at the time of commencement of the caseload trial.

Team midwifery involves a small team of midwives (often between four and ten) who provide care to a group of women (often at ‘low risk’ of complications, but some teams include women at ‘higher risk’) throughout pregnancy and birth. In some schemes, postnatal and/or domiciliary care may also be provided by the team midwives. There may be an ‘on call’ aspect for the midwives involved, but this is not usually the case in the Australian context. There is generally medical input for reviews and consultation. The aims of team midwifery are to facilitate continuity of care, increase choice for women and increase midwives’ involvement and autonomy in maternity care. In practice, the continuity achieved is limited by the number of midwives in the team, with an increased number of midwives decreasing the opportunity for continuity, but increasing the flexibility within individual midwives’ rosters [9]. Team midwifery care is associated with reduced instrumental vaginal births [4], decreased interventions during labour including induction [4,34], augmentation [4], analgesic use [4] and episiotomy [35,36], decreased caesarean sections [37,38] and satisfaction for women [36,39,41], with no statistically significant differences in perinatal morbidity or mortality [4,14]. Working in team midwifery models has been shown to increase midwives’ satisfaction although the model often takes time to be accepted, and the views of other care providers may impact on the team midwives, who in turn feel better as they perceive more support from their colleagues [19,42-44].

Caseload midwifery (also often known as Know Your Midwife or one-to-one midwifery) developed from team midwifery in an attempt to achieve care by a known caregiver [45], which the team approach is at times unable to provide. The major underlying philosophy of caseload is one of ‘continuity of carer’. The model provides the opportunity for a relationship to form between each individual woman and her primary caregiver (a midwife). This midwife, with one or two ‘back-up’ midwives, provides antenatal, labour, birthing and postnatal care. Each midwife has her own ‘caseload’ of women, and cares for approximately 40 to 45 women per year (or fewer if the midwife works less than full time) [33,45-48]. The midwife provides the majority of care for each woman in her caseload, and collaborates with obstetricians and other health professionals as necessary [33,9]. Caseload midwives manage their own workload but usually work in groups of two or three [9,33] or in group practices [48,49], working flexibly (including being on call for labour and birth) to provide 24 hour cover for their caseload and to ensure back up e.g. to cover leave, and time off. There is little rigorous evidence regarding the outcomes for women and infants of women receiving this type of care, with only two RCTs from the UK conducted in the early 1990s [50,51] (hence the reason the caseload trial was undertaken). The primary outcomes of this trial will be reported elsewhere.

### Case study one: an RCT of team midwifery for women at low risk of complications

One thousand women were recruited to the team midwifery trial between February 1996 and November 1997. Eight midwives were recruited from volunteers among the existing midwifery staff in the hospital, and team midwifery care was provided following the same clinical protocols and guidelines as standard care. The focus of care provision was antenatal and intrapartum care and a team midwife was rostered on the birth suite for each of the three shifts every day. When no team woman was in labour, the midwife cared for other women outside the team, a relatively common approach for team midwifery models [19]. Each midwife had on average one shift per week in the antenatal clinic where she saw only pregnant women enrolled in team midwifery. Occasionally the team midwives worked on the postnatal ward, but not often enough to provide continuity of care. Instead, they made follow up visits to the team women during their postnatal stay.

All included women had given birth by June 1998 and data collection was completed by September 1998. The trial found that team midwife care was associated with increased satisfaction with care [15], with no differences in birthing outcomes. These outcomes and conclusions were fed back to the hospital management and midwives as soon as available after completion of data collection. Team midwives were interviewed near the beginning of the trial and when it finished. No other interviews or data collection were undertaken.

The team midwifery model (as tested) ceased after trial completion. A team midwifery model did continue, but major changes were made, including significant changes in midwives rostering, resulting in less time in birthing suite and increased postnatal shifts. Only two of the eight midwives chose to remain working in the revised model (one had left previously and had been replaced). The new strategy meant that midwives were rostered to work on the postnatal ward for approximately 50% of the time (with some rostered birth suite shifts), so that if a team woman came in to birth suite during a shift where there was no team midwife rostered, the team midwife working in the postnatal area needed to swap with another midwife in birth suite. Anecdotal reports from the midwives working in the model at that time indicated that this way of operating was quite difficult and caused a number of issues: it was challenging to be able to care for team women during labour and birth so continuity was significantly diluted; it was not easy to allocate team women to team midwives in the postnatal area; organisational constraints did not allow team midwives to self-manage; and there were conflicting responsibilities, e.g. having to be in charge of birth suite, yet needing to care for a team woman (personal communication with three former team midwives, August 2010). The altered model was maintained with minor changes for approximately three years then ceased, after which there was no team midwifery model for some time, then a third and finally a fourth (further altered) variation of the team model was introduced. Each of the iterations of the team model that followed were an attempt to ‘get it right’ by the managers at the time, and were not aligned with the model as trialled or as described in the published literature. Rather, variations were made in attempt to keep a continuity option available to both midwives and women, as well as to achieve the right balance for the model to ‘work’ in the organisation (previous midwifery manager, personal communication, September 2010). Different options of continuity were tried and the model was offered to women of varying risk status.

There was no systematic exploration of midwives’ views of working in the models post RCT, or of the views of other stakeholders such as non-team midwives, managers and obstetric staff during or after completion of the team RCT, nor during the subsequent iterations of the team model. Therefore it is not possible to draw conclusions about why the original evaluated model was not sustained. There may have been factors such as a lack of management support, inadequate support for the team midwives, lack of engagement with or support from the obstetric staff, a lack of interest by midwives to work in the model, or concerns regarding the cost of the model. Although none of these things are known, the reasons are likely to be multifactorial. We propose that having the framework of the Normalisation Process Model/Theory or another sociological model may have assisted in identifying which of these many factors was perceived to be important in the implementation and sustainability of the model.

### Case study two: an RCT of caseload midwifery for women at low risk of complications

Eight years later, caseload midwifery was implemented in RCT conditions at the Women’s (COSMOS - COmparing Standard Maternity care with One to one midwifery Support; Australian New Zealand Clinical Trials Registry ACTRN012607000073404) [33]. One of the investigators (an author on this paper, DF) worked at both the university leading the grant application and evaluation as well as at the Women’s, and was involved in the intervention design and implementation as well as in providing ongoing support for the model.

Midwives already employed at the Women’s were offered first preference to work in the caseload model, then external advertising was used to fill further vacancies (to a total of approximately 12 full time equivalent positions). In the first 12-18 months of operation it was difficult to fill all the available staff positions, although this changed towards the end of the recruitment phase of the trial.

Recruitment of 2314 women to the trial took place from September 2007 to June 2010, with the last birth in December 2010 (primary outcomes reported elsewhere). Similar data collection methods and tools were used to evaluate the views, experiences and outcomes of the women who participated in the COSMOS trial to those used in the team trial. In addition, qualitative and quantitative data on the midwives’ experiences of working in the model have been collected. However, in contrast to the team midwifery trial, a framework for the investigation of the implementation of caseload in this setting was developed using the Normalisation Process Model [29].

Murray et al. [52] provide a description of a number of ways that Normalisation Process Theory can be used to guide researchers in conducting trials that encompass complex interventions, including the use of the theory to guide the development of the intervention, to evaluate interventions, or to guide the implementation of a complex intervention. Although Murray and colleagues [52] recommend the use of the Normalisation Process Theory to guide trials from the outset, the clinical (primary) outcomes of the COSMOS trial were conceptualised and planned much earlier, and were not considered within this theoretical context. The Normalisation Process Model *was* used in the context of the COSMOS trial to frame the evaluation of the caseload model’s implementation, as opposed to informing earlier aspects of the trial such as recruitment processes or the design of the intervention. That is, the Normalisation Process Model provided the framework to understand the implementation of caseload midwifery into clinical practice at the Women’s.

The research team worked with Carl May, the lead author of Normalisation Process Model, to consider what data collection methods and tools would best capture data to enable us to understand caseload implementation in terms of the four constructs of the Normalisation Process Model. Survey and interview questions specific to the project were thus designed to reflect the four constructs in the implementation of the new model of care.

Pre- and post-implementation surveys were undertaken with midwives working in COSMOS and in standard care, and the caseload midwives were interviewed soon after they commenced working in the model and again after the model had been functioning for two years to explore their views and experiences. Any midwife leaving the model during the trial period was invited to be surveyed and interviewed again at their time of resignation. An examination of other issues pertaining to the implementation and sustainability of the model was also built into the evaluation, including comprehensive economic evaluation; interviews with internal and external key stakeholders (managers, medical staff, representatives from industrial, professional and consumer organisations) and reconciliation of time spent on tasks in the new role.

## Discussion: can theory help?

The two case studies point to an important issue in researching and implementing new models of care – how can we account for the organisational factors that may contribute to whether a new model is adopted or not? In the case of team midwifery, the evidence suggested that this would be a useful innovation for maternity care, but it was not adopted as implemented during the trial; it was reconfigured quite significantly, disbanded soon after, then followed by yet a third iteration a few years after that.

The UK Medical Research Council (MRC) framework for researchers investigating complex interventions aims to ensure that the nature of complex interventions is fully understood and that researchers adopt appropriate methods of investigation. Developed in 2000, this framework lists complex intervention research as needing to pass through five phases in order to achieve long term implementation. The first phase of the MRC framework is the explicit use of theory: ‘A good theoretical understanding is needed of how the intervention causes change, so that weak links in the causal chain can be identified and strengthened’ [26]. This is particularly important given that the components of a complex intervention are interlinked and that the organisational or social context in which they are researched itself forms part of the intervention. Theoretical knowledge can inform our understanding of program continuation, an outcome that may have been expected after the team midwifery trial. Institutionalising, normalising [30,31] and routinising [53] are all focused on how an intervention can become part of the everyday framework of the intervention site. These are valuable terms in capturing how practices or innovations that are initiated become part of the day to day operations of a program by focusing on the work required to integrate and embed changes in practice.

In the early phases of the caseload midwifery trial the Normalisation Process Model was identified as a sociological model that might provide an appropriate theoretical framework to underpin the aspects of the research related to the implementation of the new model into practice. The research team wanted to move beyond the simple notion of ‘evidence of benefit’ and to think about how changes made to service provision became either accepted or rejected within the organisation. Although the Normalisation Process Model was not used in the design of the RCT itself, it did underpin and provide a framework for a large part of the non-clinical aspects of the evaluation, and focused attention on other aspects such as the adoption of a new role for midwives and the support of key stakeholders in the organisation.

Drawing on the original description of the four constructs of the Normalisation Process Model as described earlier, the following is a brief description of our initial conceptualisation of how the four constructs might apply to the implementation and evaluation of the caseload midwifery model. 1. *Interactional workability* could be identified as a shared understanding of the new role, particularly by those who receive the service. If caseload was to be ‘normalised’ the women accessing the service would need to perceive this as a model of care that they can access for safe and satisfactory care, with a clear understanding of the role of the caseload midwives. 2. The construct of *relational integration* could reflect the peer and professional perceptions of the new model of care. Caseload could become normalised if the care provided by caseload midwives was seen to be safe, and if the caseload midwives were seen to have the skills needed to perform their role, and that they were able to assume the professional responsibilities of their role. 3. *Skill set workability* is a construct that reflects the organisational division of labour - who should do the work? For caseload to be normalised in the organisation there would need to be a clear articulation of the responsibilities of caseload midwives in relation to other staff, including midwives in standard care and medical staff. 4. The final construct within the Normalisation Process Model, *contextual integration* could be reflected by change within the organisation to ensure the availability of the resources appropriate to provide the model and a place within the organisation for the model to ‘fit’. Caseload could become normalised if it were supported by management as a component of cost effective services offered by the organisation.

We therefore embedded our understanding of the four constructs of the Normalisation Process Model in many aspects of our investigation of the implementation of the caseload midwifery model, using both qualitative and quantitative methods. The midwives’ surveys included questions reflecting the four constructs of the model, and explored how the midwives interpreted the organisational acceptance of the new role in both clinical practice and organisational structure. Semi-structured interviews aimed at obtaining a more in-depth exploration also drew on the four constructs to investigate the change of the ‘work’ that was involved in being a caseload midwife. The interviews explored a number of issues relating to the model, including motivation to work in the role, how the work differs from standard midwifery ‘work’, the processes of implementation and the functioning of the new model of care. A similar interview structure was used with the internal and external key stakeholders. A full description of the development and piloting of these data collection tools will be provided elsewhere, but Additional file 1 provides examples of the final survey questions and topic areas for discussion in the semi-structured interviews.

The development of data collection tools that reflected the constructs of the Normalisation Process Model provide information on the perceptions of midwives, as well as those of internal and external key stakeholders, in an attempt to identify how the constructs impacted on the implementation and embedding (or not) of the model within the organisation. Incorporating data collection on the four constructs from sources within and outside the model, at two time points (at the model’s commencement and after two years) will enable us to form a conceptual understanding of how these constructs influence both implementation and ongoing sustainability of the model within the organisation. It may also show if any of the constructs has a greater weighting on the normalisation of the model in this setting. That is, the use of this theoretical model will deepen our understanding of which factors contribute to the legitimacy of an intervention and thus the likelihood that it will be sustainable.

### Using the Normalisation Process Model to reflect retrospectively

Once the Normalisation Process Model has been applied in this way in the RCT of caseload midwifery, it may also be able to be used to reflect retrospectively on why ‘successful’ trials, such as the team midwifery RCT, are not subsequently implemented and ‘normalised’ as part of maternity care. While it may not be appropriate to apply a theoretical approach to the consideration of implementation of the original team midwifery model, we have used the four constructs of the Normalisation Process Model to help us reflect on possible reasons that the team model did not ‘succeed’ at the completion of the RCT despite the positive trial outcomes.

In terms of the *interactional workability* (how the work is ‘enacted’ by the people doing it), the model was no longer able to be enacted in the way it had been previously. Major changes were made to the organisation of the post RCT team midwifery model. The work of the team midwives changed significantly – they had more time allocated to postnatal care than intrapartum care. Women’s expectations that care would be provided by a team midwife, particularly during labour and birth may have been affected by the new requirement to staff the postnatal area.

Likewise, given the major change in the structure of the model, it is possible that the *relational integration* (how the work was understood) was compromised. It may be that when an RCT such as this is in progress a somewhat ‘artificial’ environment may be created, where there are ‘rules’ around what needs to be in place. Thus when the RCT ends the perceived ‘rules’ no longer apply and make the model as tested more vulnerable to change. Despite the model being implemented for three years (that is in the RCT context), the new model may have been poorly understood in the organisational context and hence inadequately integrated or normalised. The required movement of staff between areas mid-shift may have led to confusion relating to the structure of the model within the organisation.

This revised way of operating may have led to less stability or less clarity regarding the division of labour between team midwives and those in standard care as reflected in the construct of *skill set workability*. The alterations to the model may have made the issue of ‘who should do the work’ less clear, especially when the priority of providing intrapartum care was made more difficult by postnatal rostering. Midwives in the original team model self selected to work in the program and were mostly very senior, experienced birth suite clinicians. It could be argued that it was viewed as a prestigious opportunity to work in the new way. The post trial model significantly increased postpartum care provision with a decrease in labour and birth care for team midwives. Postnatal care is often considered the poor cousin of maternity care [54] and may at the time have been viewed less favourably, and not in alignment with the team midwives’ view of where their skills were best suited.

It is possible with the changes to the team model that the place within the organisation where the model had been designed to ‘fit’ no longer existed. That is, that the *contextual integration* was no longer present. The ongoing changes to the model after completion of the trial may have raised questions as to the commitment of the organisation to offer a place for this model and may have diminished confidence in the organisational commitment to the team model of care. Again, we stress that these are reflections only.

## Conclusion

Although it may be difficult to look retrospectively at interventions and apply a theoretical approach (which is why we can only speculate about team midwifery), the strength of using the Normalisation Process Model/Theory is that it enables identification of the factors to be taken into account when planning and implementing complex interventions. 'It focuses attention on the work that people need to do to implement and integrate new health care practices’ [55].

The use of theory can contribute to a clearer idea about how we can understand issues around implementation and sustainability, particularly when research is framed with a clear focus on what factors might be important. The Normalisation Process Model has provided a framework within the COSMOS trial to examine some of these issues prospectively, both through the evaluation research design (relating to the implementation of the model of care into practice) and analysis of findings. Organisations may use the evidence from the trial findings to guide implementation strategies, ensuring that constructs that have been identified as important for the model’s sustainability are encompassed in implementation strategies. In addition, future trials of models of care may benefit from using the Normalisation Process Theory not only to understand implementation, but to guide trial design and development of the intervention, as suggested by Murray et al. [52].

The value of having information on not only the effectiveness of new models of care, but on how and why models do or don’t work within organisations provides a new way of considering complex interventions. Organisations considering the introduction of new models invest significant time, energy and resources, particularly when, as in the case studies used for illustration in this paper, they are part of trials. The ‘answer’ as to the effectiveness of a model lies not only in clinical effectiveness, but within the organisation’s ability to find an ongoing ‘space’ for the change.

## Competing interests

The authors declare that they have no competing interests.

## Authors' contributions

All authors were responsible for the conceptualisation of the paper. DF drafted the original manuscript and all authors then contributed to the development and completion of the paper. All authors read and approved the final version.

## Supplementary Material

Additional file 1 **Table:** Examples of questions/statements to explore four constructs of Normalisation Process Model in the caseload trial. Click here for file
