# Supplementary material for: Exploring implementation and sustainability of models of care: can theory help?
Source: BMC Public Health. 2011 Nov 25;11(Suppl 5):S8. doi: 10.1186/1471-2458-11-S5-S8 (PMC3247031; doi:10.1186/1471-2458-11-S5-S8)
Supplement: Additional file 1 — Table: Examples of questions/statements to explore four constructs of Normalisation Process Model in the caseload trial. [file 1471-2458-11-S5-S8-S1.doc]

# Additional file 1

**Table 1:**

Examples of questions/statements to explore four constructs of Normalisation Process Model in the caseload trial

| **Construct** | **Example of survey and interview questions/statements in exploring midwives views** |
| --- | --- |
| **Interactional workability**  **(How the work is enacted by the people doing it)** | Survey: Caseload will make it easier for midwives to work collaboratively with women  Interview: What difference do you think this model of care might make to women? |
| **Relational integration (How the work is understood within the networks)** | Survey: Caseload will improve the way that midwives’ expertise is understood by others.  Interview: How do you think those working in caseload are perceived? |
| **Skill set workability (The place of work in a division of labour)** | Survey: Caseload midwives will need to have a different level of skill and competence than non-caseload midwives.  Interview: Has caseload changed the type of work that you do? |
| **Contextual integration (Organisational sponsorship and control of work)** | Survey: The benefits of caseload are likely to be outweighed by the resources needed to provide care within the model  Interview: How do you see the role of management in this model? |
